# Piperine Improves Lipid Dysregulation by Modulating Circadian Genes *Bmal1* and *Clock* in HepG2 Cells

**DOI:** 10.3390/ijms23105611

**Published:** 2022-05-17

**Authors:** Weiyun Zhang, Chi-Tang Ho, Muwen Lu

**Affiliations:** 1 Guangdong Provincial Key Laboratory of Nutraceuticals and Functional Foods, College of Food Science, South China Agricultural University, Guangzhou 510642, China; weiyunzhangg@gmail.com; 2 Department of Food Science, Rutgers University, New Brunswick, NJ 08901, USA; ctho@sebs.rutgers.edu

**Keywords:** piperine, circadian clock, lipid accumulation, *Bmal1* gene, *Clock* gene

## Abstract

Metabolic disorders are closely associated with the dysregulation of circadian rhythms. Many bioactive components with lipid metabolism-regulating effects have been reported to function through circadian clock-related mechanisms. As the main pungent principle of black pepper, piperine (PIP) has been demonstrated to possess anti-obesity bioactivity by affecting hepatic lipid metabolism-related factors. However, whether the circadian clock genes *Bmal1* and *Clock* are involved in the protective effect of PIP against lipid metabolism disorders remains unknown. In this work, oleic acid (OA) induced lipid accumulation in HepG2 cells. The effect of PIP on redox status, mitochondrial functions, and circadian rhythms of core clock genes were evaluated. Results revealed that PIP alleviated circadian desynchrony, ROS overproduction, and mitochondrial dysfunction. A mechanism study showed that PIP could activate the SREBP-1c/PPARγ and AMPK/AKT-mTOR signaling pathways in a *Bmal1*/*Clock*-dependent manner in HepG2 cells. These results indicated that *Bmal1* and *Clock* played important roles in the regulating effect of PIP on hepatic lipid homeostasis.

## 1. Introduction

**Figure 1:**
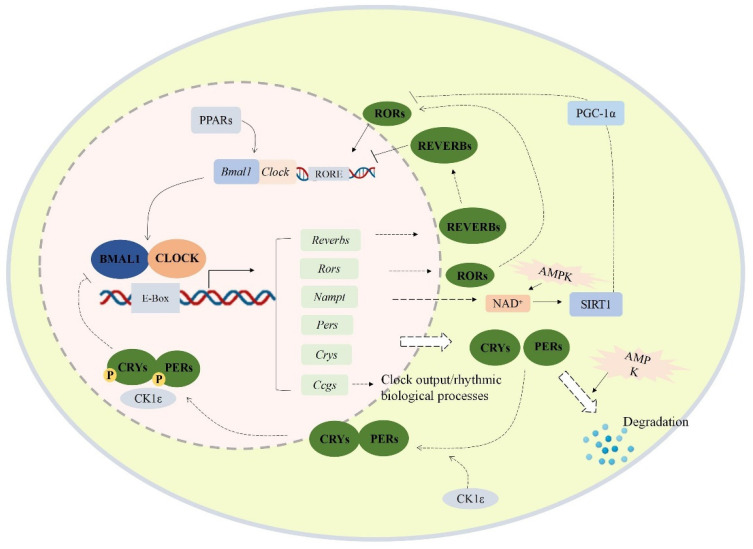
Transcriptional/translational feedback loop of mammalian circadian genes. The cellular circadian system contains an autoregulatory transcriptional/translational feedback loop. PERs/CRYs and CLOCK/BMAL1 loop are the core of the cellular oscillator. CLOCK and BMAL1 form heterodimers and translocate into the nucleus, then bind to the E-box response element of the upstream promoter region of the clock control gene to activate the transcription of the target gene. Meanwhile, the PERs and CRYs proteins also form heterodimers and translocate into the nucleus to inhibit the transcription of *Clock* and *Bmal1*. Besides, the expression of *Nampt*, *Rev-erbs*, *Ppars*, *Ccgs*, and *Rors* is also promoted by CLOCK/BMAL1 heterodimer. Abbreviations: BMAL1 (aryl hydrocarbon receptor nuclear translocator like 1 proteins), *Bmal1* (aryl hydrocarbon receptor nuclear translocator like 1), CLOCK (circadian locomotor output cycles kaput proteins), *Clock* (circadian locomotor output cycles kaput), PERs (period proteins), *Pers* (period genes 1/2/3), CRYs (cryptochrome proteins), *Crys* (cryptochrome genes 1/2), RORs (retinoid-related orphan receptors), Reverbs (reverse erythroblastosis virus α/β), Ccgs (other clock-controlled genes), SIRT1 (silent information regulator 1), Nampt (nicotinamide phosphoribosyltransferase gene), RORE (ROR response element), PGC-1α (proliferator-activated receptor gamma coactivator 1-α), AMPK (AMP-activated protein kinase), PPARs (peroxisome proliferator-activated receptors).

The circadian clock is a molecular pacemaker in the human body that regulates 24-h physiological and behavioral processes [1,2]. It is controlled by the central clock system located in the hypothalamus suprachiasmatic nucleus (SCN), which receives environmental light signals through the retinohypothalamic tract [3]. As shown in Figure 1, cell-autonomous mammalian circadian rhythm oscillators function through an autoregulatory transcriptional/translational feedback loop (TTFL), which is driven by activators (CLOCK and BMAL1) and inhibitors (PER and CRY) [4]. Heterodimers of the CLOCK and BMAL1 proteins could bind to the E-box of CACGTG and activate the transcription of cryptochrome (CRY) and period (PER) genes [5]. The PER and CRY proteins gradually accumulate and form a heterodimer to inhibit CLOCK/BMAL-mediated transcription of the *Per* and *Cry* genes [6].

Multiple studies have shown that the misalignment of circadian rhythms is associated with various metabolic disorders, such as obesity, cancer, inflammatory response, insulin resistance, and diabetes [5,7,8,9,10]. Many phytochemicals with metabolism-modulation effects have been proved to restore normal circadian rhythm in cell models and animal models, such as tea polyphenols, resveratrol, cichoric acid, and capsaicin [11,12,13,14,15,16]. Guo et al. reported that cichoric acid could attenuate the lipid metabolism dysfunction by modulating the circadian gene *Bmal1* expressions in hepatocytes [11]. Resveratrol could also normalize the liver circadian rhythmic disorder of lipid metabolism induced by a high-fat diet in male C57BL/6 mice [17]. In our previous work, capsaicin ameliorated the circadian disruption triggered by glucosamine treatment in HepG2 cells, suggesting that the circadian clock played an important role in metabolism homeostasis regulated by bioactive food ingredients [12].

Piperine (1-piperoylpiperidine, PIP), the major pungent alkaloid constituent of Piper nigrum (black pepper), has been reported to possess lipid metabolism-improving effects both in vitro and in vivo [18]. However, whether circadian clock regulators are involved in the modulation process of lipid metabolism by PIP remains unknown. In this work, we examined the intervention effect of PIP on circadian disorders and lipid accumulation induced by oleic acid (OA) in human hepatoma HepG2 cells. The role of circadian clock genes *Bmal1* and *Clock* in the regulation process of PIP on lipid accumulation was also investigated.

## 2. Results and Discussion

### 2.1. Preventive Effects of PIP on OA-Induced Lipid Accumulation in HepG2 Cells

**Figure 2:**
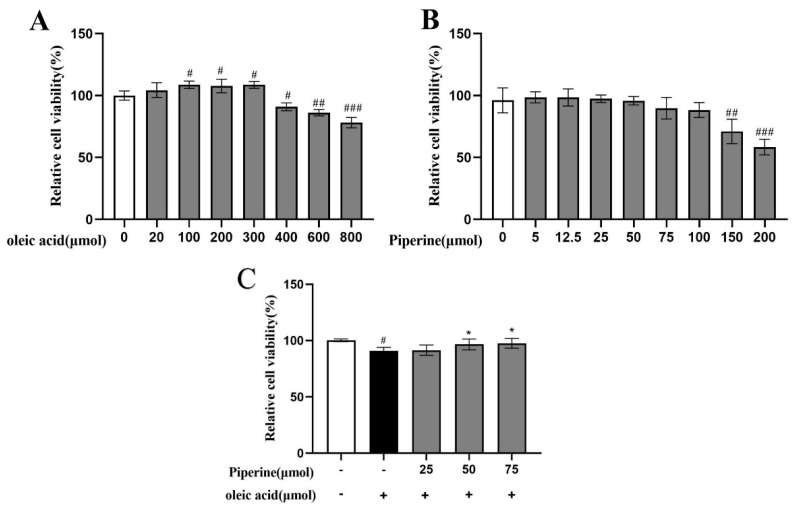
Effect of PIP and OA on HepG2 cell viability measured by MTT assay. (**A**) Relative viability of HepG2 cells treated with different concentrations of OA. (**B**) Relative viability of HepG2 cells treated with different concentrations of PIP. (**C**) Relative cell viability treated with 25, 50, 75 μM PIP and co−treated with/without 400 μM OA. The results are expressed as the means ± SD, *n* ≥ 3. ^#^ *p* < 0.05, ^##^ *p* < 0.01 ^###^ *p* < 0.001 versus control group, * *p* < 0.05versus OA group.

The effect of different concentrations of PIP and OA on the cell viability of HepG2 cells was assessed using the MTT assay. As shown in Figure 2A, with the increase in the OA concentration from 20 to 300 μM, the cell viability increased from 104.35 ± 5.95 to 108.63 ± 2.79% (*p* < 0.001), indicating a cell proliferation-promoting effect of OA at low concentrations. After the concentration of OA reached 400 μM, cell viability started to decrease. At PIP concentrations of 400 to 800 μM, the viability reduced from 90.94 ± 3.16 to 78.15 ± 4.19% (*p* < 0.001), suggesting that 400 μM was the critical concentration of OA to perform the cell study with low cytotoxicity.

According to Figure 2B, HepG2 cells were treated with 0, 5, 12.5, 25, 50, 75, 100, 150, and 200 μΜ PIP. At concentrations of 0 to 25 μΜ, the PIP showed no toxic effect on cell viability. As PIP concentration gradually increased from 25 to 75 μΜ, the cell viability was decreased from 97.40 ± 3.02 to 89.77 ± 8.72% (*p* < 0.001) in a dose-dependent manner. As concentration further accumulated, the cell death rate increased significantly to 52.63 ± 6.35% (*p* < 0.001), indicating that PIP at a concentration below 75 μΜ was critical to maintaining normal cell activity. As shown in Figure 2C, PIP (50, 75 μM) treatment for 24 h could significantly increase the viability of HepG2 cells from 90.85 ± 3.16% to 96.65 ± 4.69% (50 μM) and 97.96 ± 4.33% (75 μM) (*p* < 0.05) respectively compared with the OA-treated group, suggesting that PIP efficiently reversed OA-induced decrease of cell viability. In this study, HepG2 cells treated with OA and PIP were examined based on the effect of PIP on lipid levels, cell redox states, and mitochondrial functions disrupted by OA.

**Figure 3:**
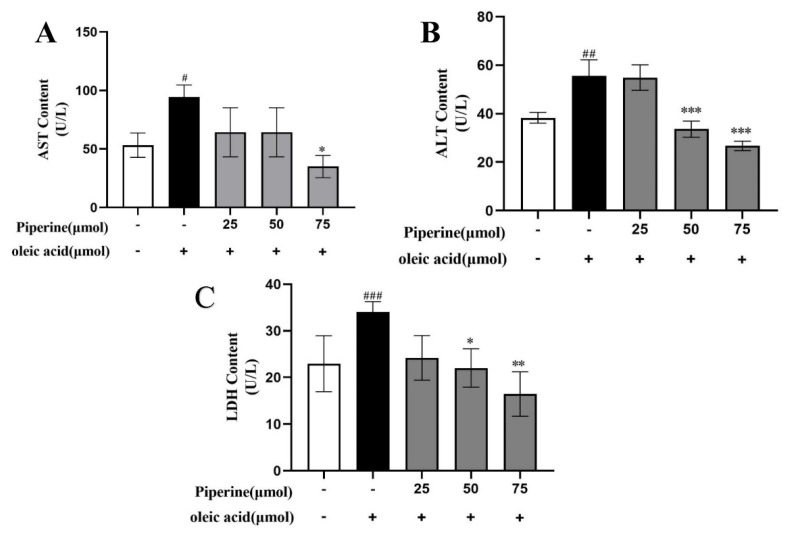
Effects of PIP on OA−induced liver function in HepG2 cells. (**A**) Aspartate aminotransferase (AST) level at wavelength of 510 nm (**B**) Alanine aminotransferase (ALT) level at wavelength of 510 nm. (**C**) Lactate dehydrogenase (LDH) level at wavelength of 450 nm. Results were expressed as the means ± SD, *n* ≥ 3. ^#^ *p* < 0.05, ^##^ *p* < 0.01 ^###^ *p* < 0.001 versus control group, * *p* < 0.05, ** *p* < 0.01, *** *p* < 0.001 versus OA group.

To evaluate the protective effects of PIP on OA-induced hepatotoxicity in HepG2 cells, the levels of ALT, AST, and LDH were examined. As the notable signs of cellular damage and integrity of the liver cell membrane, AST, ALT, and LDH were released into the serum when cells were damaged or necrotic [19]. Compared to the untreated group, OA treatment significantly increased the activities of ALT, AST, and LDH, which indicated that hepatocyte damage was induced by OA. As shown in Figure 3, the addition of PIP (50, 75 μM) decreased the level of ALT from 55.61 ± 6.56 U/L to 33.63 ± 3.32 U/L and 26.67 ± 1.39 U/L (*p* < 0.01), respectively. PIP (75 μM) treatment also reduced the content of AST (from 94.53 ± 10.16 U/L to 35.05 ± 9.39 U/L (*p* < 0.05)) and LDH (from 34.17 ± 2.19 U/L to 16.14 ± 4.77 U/L (*p* < 0.01)), demonstrating that PIP at 50 and 75 μM could significantly inhibit liver cell damage induced by OA.

### 2.2. Inhibitory Effects of PIP on OA-Induced Lipids Accumulation in HepG2 Cells

**Figure 4:**
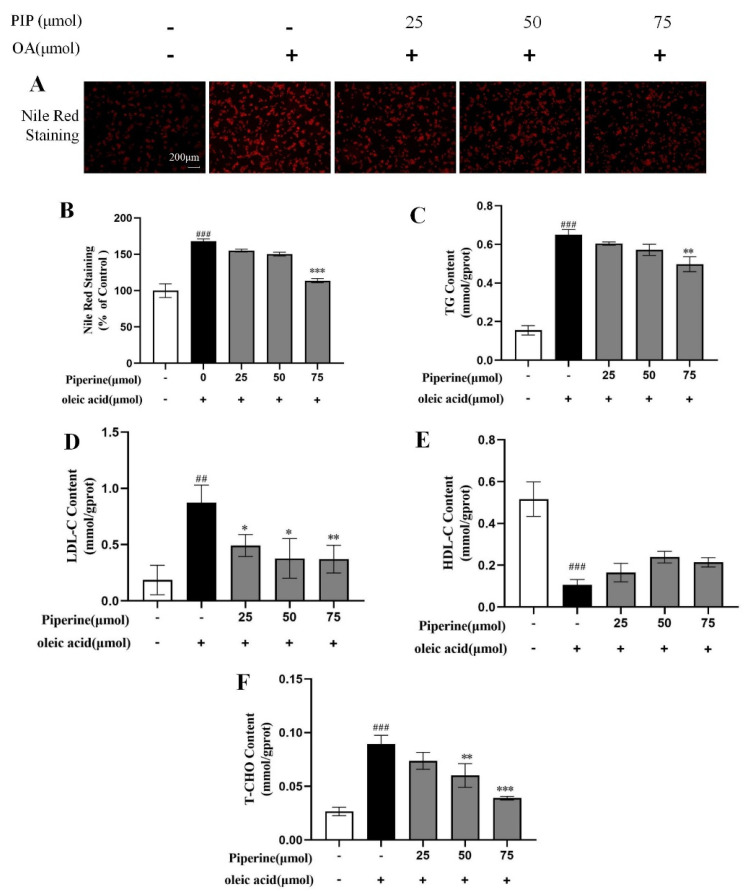
Effects of PIP on OA-induced lipids levels of HepG2 cells. HepG2 cells were treated with different concentrations of PIP (25, 50, and 75 μM) and co-treated with/without OA (400 μM) for 24 h. (**A**) Nile red staining was observed by an inverted fluorescence microscope. (**B**) The relative fluorescence intensity of Nile red staining. (**C**) Total triglyceride (TG). (**D**) LDL cholesterol (LDL-C) (**E**) HDL cholesterol (HDL-C). (**F**) Total cholesterol (TC). Results were expressed as the means ± SD, *n* ≥ 3. ^##^ *p* < 0.01 ^###^ *p* < 0.001 versus control group, * *p* < 0.05, ** *p* < 0.01, *** *p* < 0.001 versus OA group.

Nile red staining method was used to quantitatively measure lipid levels and to qualitatively observe lipid distribution across cells [20,21,22]. As shown in Figure 4A,B, a large number of red-stained lipid droplets could be observed with increased intensity of Nile red fluorescence by 68.07 ± 2.07% (*p* < 0.001) in the OA-treated group compared with the control group, showing that OA treatment-induced intracellular lipid accumulation in HepG2 cells. 25 μM PIP decreased the Nile red fluorescence intensity by 13.07 ± 1.31% compared to the OA group. With the increase in the PIP concentration from 25 to 75 μM, the inhibition effect was further enhanced from 13.07 ± 1.31 to 54.3 ± 2.13% (*p* < 0.001), indicating that PIP could suppress the OA-induced lipid accumulation in a dose-dependent manner.

TG, TC, LDL-C, and HDL-C contents in hepatocytes were commonly used to measure the lipid levels [23]. As presented in Figure 4C–F, OA elevated cellular TG, TC, and LDL-C contents and reduced the level of HDL-C compared to the control group, suggesting that OA disrupted the lipid metabolism homeostasis in HepG2 cells. It was reported that PIP could regulate lipid levels by enhancing cholesterol efflux, reducing cholesterol uptake, and leading to a reversal of hepatic homeostasis induced by HFD [24]. In our experiment, after PIP treatment, TG, TC, and LDL-C contents were reduced dose-dependent, showing that PIP ameliorated the accumulation of lipids in HepG2 cells.

### 2.3. Effects of PIP on OA-Induced Mitochondrial Dysfunction and ROS Overproduction in HepG2 Cells

**Figure 5:**
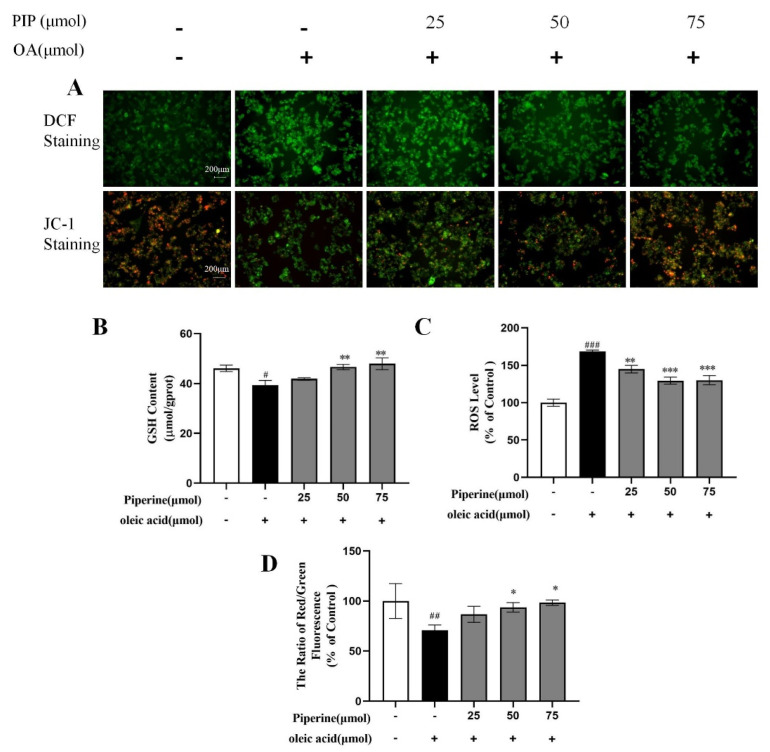
Effects of PIP on OA-induced intracellular redox imbalance and mitochondrial dysfunction in HepG2 cells. (**A**) DCF staining and JC−1 staining observed by inverted fluorescence microscope. (**B**) Glutathione (GSH) level at a wavelength of 405 nm. (**C**) Reactive oxygen species (ROS) measured using reactive oxygen species assay kit. (**D**) Mitochondrial membrane potential (MMP) was reflected as the ratio of red/green using fluorescence microscopy. The results were expressed as the means ± SD, *n* ≥ 3. ^#^ *p* < 0.05, ^##^ *p* < 0.01 ^###^ *p* < 0.001 versus control group, * *p* < 0.05, ** *p* < 0.01, *** *p* < 0.001 versus OA group.

Oxidation of fatty acids mainly occurs in the mitochondria, leading to the production of a considerable amount of ROS [25]. Studies revealed that oxidative damage could be induced through the overgeneration of ROS and reducing cellular GSH levels, consequently resulting in lipid accumulation and even cell apoptosis [26,27]. As an important tripeptide reductant that can protect lipid from oxidation and maintain cellular redox status [28], GSH level was measured and shown in Figure 5B. The OA-treated cells exhibited a significant decrease in GSH content by 6.75 ± 1.30% compared to control groups, indicating that OA caused oxidative damage in HepG2 cells. After PIP treatment, GSH level was recovered dose-dependently, which suggested that PIP could modulate redox status and prevent oxidative damage in HepG2 cells. The ROS production level measured by fluorochrome dichloroflurorescin diacetate (DCFH-DA) was shown in Figure 5C. After OA treatment, the relative ROS level increased from 100 to 168.81 ± 1.79% (*p* < 0.001), showing that OA induced the overproduction of ROS in HepG2 cells. Different concentrations of PIP (25, 50, 75 μM) decreased ROS levels to 144.83 ± 5.14, 130.04 ± 4.17, and 129.48 ± 6.13% (*p* < 0.001), respectively, suggesting that PIP inhibited the ROS overproduction stimulated by OA. These results revealed that PIP supplementation alleviates oxidative damage by reducing the ROS levels and increasing GSH levels of HepG2 cells in a dose-dependent manner.

As an important marker of cellular damage, the mitochondrial membrane potential (MMP) in HepG2 cells was measured by JC-1 staining to further evaluate the protective effect of PIP on mitochondrial functions [29,30]. JC-1 could form complexes with intense red fluorescence spontaneously in cells under high MMP, and form monomers with green fluorescence in unhealthy cells with low MMP [31]. As shown in Figure 5D, compared to the control group, the treatment with OA for 24 h decreased the MMP from 100 to 70.51 ± 5.26% (*p* < 0.01), indicating that OA caused oxidative damage in mitochondria. The MMP levels were increased to 86.74 ± 8.09, 93.69 ± 4.78, and 98.41 ± 2.70% (*p* < 0.05) by PIP (25, 50, 75 μM) treatment, respectively, which meant that PIP could reverse the mitochondrial dysfunction induced by OA in a dose-dependent manner. Overall, PIP was essential in preventing oxidative damage in HepG2 cells injured by OA.

### 2.4. Intervention Effect of PIP on Circadian Misalignment

**Figure 6:**
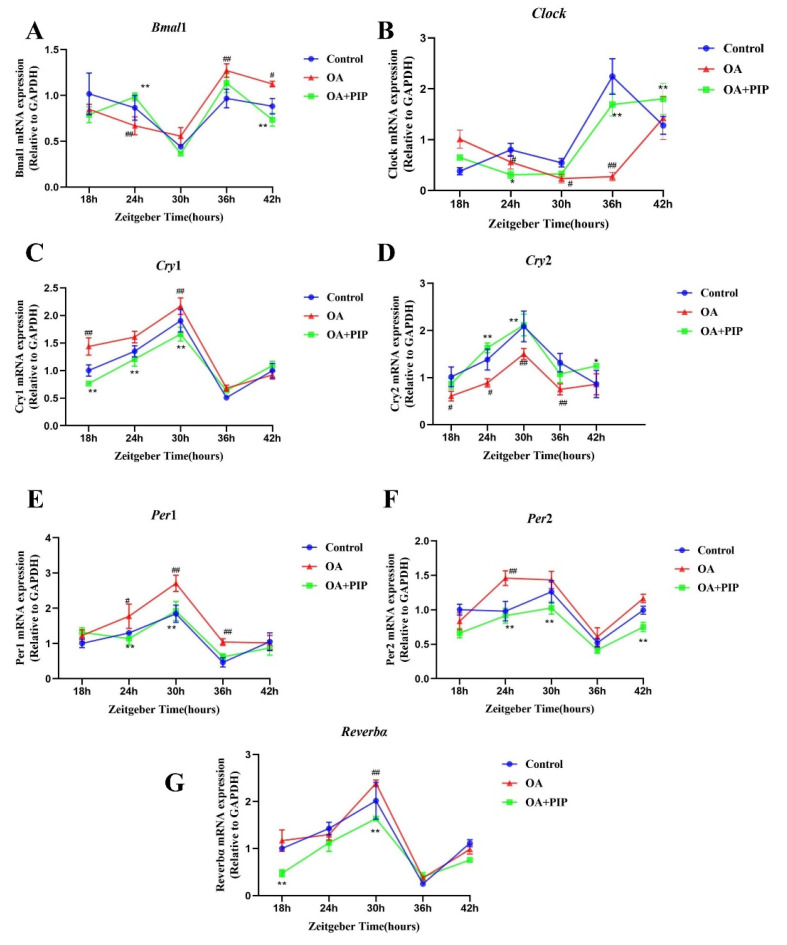
Effects of PIP on OA-induced circadian misalignment. HepG2 cells were treated with OA (400 μM) and PIP (50 μM) for 24 h. Cells then were collected for mRNA analysis at 6 h intervals between 18 h and 42 h time points. (**A**–**G**) The mRNA levels of core circadian clock genes *Bmal1*, *Clock*, *Per1*, *Per2*, Cry1, *Cry2*, and *Reverbα* in HepG2 cells. Transcript levels were measured by RT-qPCR and normalized to GAPDH. Results were expressed as the means ± SD, *n* ≥ 3. ^#^ *p* < 0.05, ^##^ *p* < 0.01 versus control group, * *p* < 0.05, ** *p* < 0.01 versus OA group.

Recent studies revealed that circadian desynchrony was closely related to the disruption of glucose and lipid homeostasis [32,33,34,35,36]. According to Li et al., the misalignment of circadian clock genes could be caused in the lipid accumulation model using HepG2 cells [13]. Similar trends were also observed in our study. The expressions of circadian clock genes in HepG2 cells were measured every 6 h to evaluate their rhythmic patterns. As shown in Figure 6, rhythmic gene expressions could be found for *Bmal1*, *Clock*, *Per1-2*, *Cry1-2*, and *Reverbα* in the control group. OA treatment-induced phase shift in *Clock* and *Per2* genes and shallowed oscillation of *Cry2* gene and increased the oscillatory amplitude of *Per1*, *Cry1*, and *Reverbα* expression levels, indicating that circadian rhythm desynchrony was triggered in the lipid accumulation model. After PIP treatment, the phase shift of the *Clock* gene and shallowed oscillation of the *Cry2* gene were ameliorated, and the intensity of oscillatory amplitude of *Per1* expression was recovered. These results demonstrated that PIP could alleviate the circadian misalignment triggered by OA in HepG2 cells.

### 2.5. PIP Attenuated OA-Induced Redox Imbalance and Mitochondrial Dysfunction via Regulating Bmal1 and Clock in HepG2 Cells

**Figure 7:**
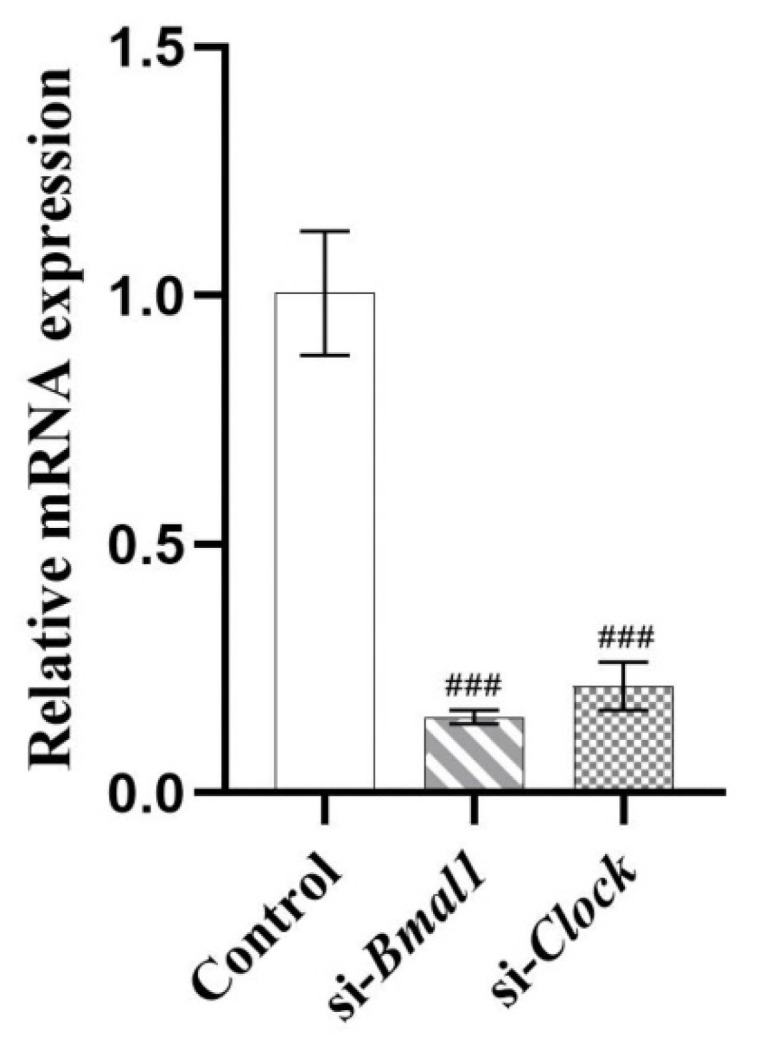
HepG2 cells were transfected with the si-*Bmal1*/si-*Clock* using Lipofectamine 2000 transfection reagent. Transcript levels were measured by RT-qPCR and normalized to GAPDH. Results were expressed as the means ± SD, *n* ≥ 3. ^###^ *p* < 0.001 versus control group.

To verify whether the circadian clock genes were involved in the modulation process of lipid metabolism by PIP, HepG2 cells were transfected with si-*Bmal1* or si-*Clock,* respectively, for 24 h. The mRNA expression of *Bmal1* and *Clock* were inhibited to 15.32 ± 1.42% and 21.46 ± 4.79% (*p* < 0.001) (Figure 7), suggesting that the siRNA transfection inhibited the RNA expressions of *Bmal1* and *Clock* genes successfully.

**Figure 8:**
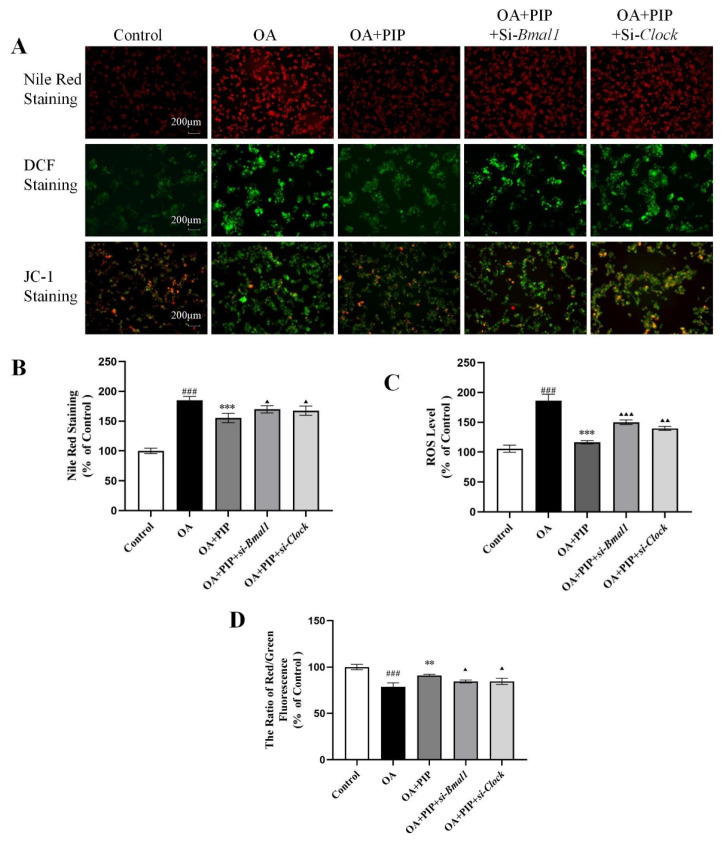
PIP attenuated OA-induced lipid metabolic disturbance, redox status imbalance and mitochondria dysfunction in a *Bmal1*/*Clock*-dependent way. (**A**) Nile Red staining, DCF staining, and JC-1 staining observed by inverted fluorescence microscope. (**B**) The relative fluorescence intensity of Nile red staining. (**C**) Reactive oxygen species (ROS) measured using a reactive oxygen species assay kit. (**D**) Mitochondrial membrane potential (MMP) reflected as the ratio of red/green using fluorescence microscopy. Results were expressed as the means ± SD, *n* ≥ 3. ^###^ *p* < 0.001 versus control group, ** *p* < 0.01, *** *p* < 0.001 versus OA group, ^▲^ *p* < 0.05, ^▲▲^ *p* < 0.01, ^▲▲▲^ *p* < 0.001 versus OA + PIP group.

According to Figure 8, the Nile Red staining results indicated that the silencing of *Bmal1* or *Clock* expression inhibited the recovery effect of PIP on lipid accumulation. The intensity of Nile red staining in the OA + PIP-treatment group was 29.60 ± 1.06% (*p* < 0.001) lower than in the OA group, whereas OA + PIP + si-*Bmal1* and OA + PIP + si-*Clock*-treatment groups showed significant 14.60 ± 1.79% and 12.18 ± 0.10% (*p* < 0.05) increase compared with the OA + PIP group, demonstrating that PIP treatment could inhibit the OA-disrupted lipid homeostasis, and knockdown of genes *Bmal1* and *Clock* suppressed this alleviation effect of PIP. As shown in Figure 8C,D, after the knockdown of *Bmal1* or *Clock* gene, the ROS level was increased from 116.31 ± 2.77% in the OA + PIP-treatment group to 149.99 ± 3.87% (*p* < 0.05) and 139.65 ± 3.81% (*p* < 0.001) in the OA + PIP + si-*Bmal1* and OA + PIP + si-*Clock*-treatment groups respectively. Similarly, the MMP was decreased from 90.99 ± 1.20% in the OA + PIP group to 84.48 ± 1.61% and 85.56 ± 3.32% (*p* < 0.01) in the OA + PIP + si-*Bmal1* and OA + PIP + si-*Clock* groups respectively. These results indicated that PIP regulated the intracellular redox imbalance and mitochondrial dysfunction via modulating the expressions of the circadian clock genes *Bmal1* and *Clock*.

### 2.6. Effects of PIP on OA-Induced Lipid Metabolism Disorder Imbalance in a Bmal1/Clock-Dependent Way

**Figure 9:**
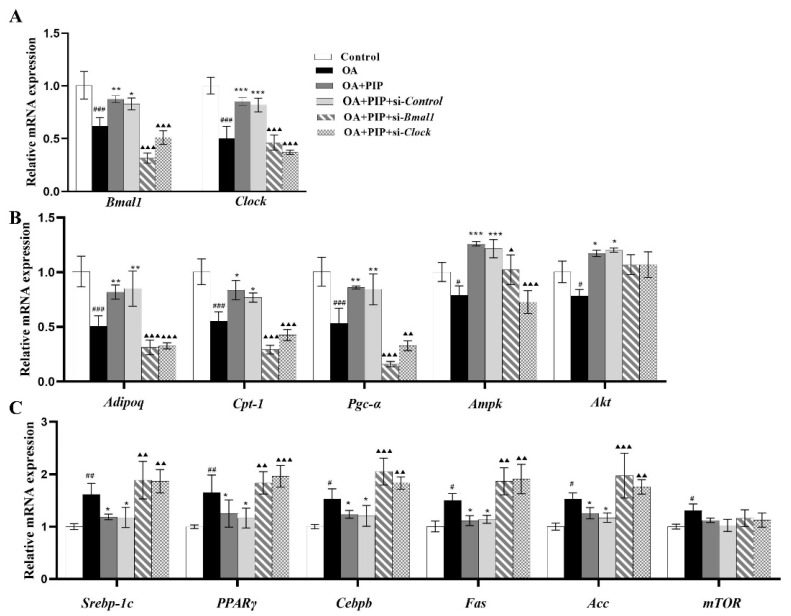
PIP ameliorated OA-induced lipid-metabolism disorder by regulating the circadian clock and lipid metabolism-related factors in mRNA levels. HepG2 cells were transfected with the si-*Control* or si-*Bmal1*/si-*Clock* for 24 h, the cultured cells treated with OA (400 μM) and PIP (50 μM) for 24 h. GAPDH was used as a loading control. (**A**) The mRNA levels of *Bmal1* and *Clock*. (**B**) The mRNA levels of *Adipoq*, *Pgc-1α*, *Cpt-1*, *Ampk*, and *Akt*. (**C**) The mRNA levels of *Srebp-1c*, *Cebpb*, *Pparγ*, *Fas*, *Acc* and *mTOR*. Results were expressed as the means ± SD, *n* ≥ 3. ^#^ *p* < 0.05, ^##^ *p* < 0.01 ^###^ *p* < 0.001 versus control group, * *p* < 0.05, ** *p* < 0.01, *** *p* < 0.001 versus OA group, ^▲^ *p* < 0.05, ^▲▲^ *p* < 0.01, ^▲▲▲^ *p* < 0.001 versus OA + PIP group.

To further explore the role of gene *Bmal1* and *Clock* played in the preventative effect of PIP on lipid metabolism disorder, core circadian genes *Bmal1* and *Clock* were knocked down in HepG2 cells. As shown in Figure 9, OA treatment increased the mRNA expression levels of circadian clock genes *Bmal1* and *Clock*, lipid metabolism-related genes sterol-regulatory-element-binding protein 1c (Srebp-1c), peroxisome proliferator-activated receptor γ (Pparγ), CCAAT enhancer-binding proteins β (Cebpb), fatty acid synthase (Fas), acetyl-CoA carboxylase (Acc) and mammalian/mechanistic target of rapamycin (mTOR), while PIP suppressed the overexpression of Srebp-1c, Cebpb, Pparγ, Fas, and Acc effectively. As an adipokine involved in the regulation of the lipid metabolism process, the RNA content of adiponectin as well as carnitine palmitoyltransferase 1 (CPT-1) and peroxisome proliferator-activated receptor gamma coactivator 1α (PGC-1α) were also evaluated to examine whether they functioned through circadian clock-involved mechanisms [37,38]. The mRNA expression levels of Adipoq, Pgc-1α, Cpt-1, AMP-activated protein kinase (Ampk), and serine-threonine kinase (Akt) in the OA group were decreased compared to the control group, which were increased after PIP treatment. These results indicated that PIP ameliorated the OA-induced gene overexpression of lipid metabolism-related factors. Additionally, knockdown of *Bmal1* and *Clock* weakened the recovery effect of PIP on most lipid metabolism-related gene expressions. Therefore, the regulation effect of PIP on the mRNA level of lipid metabolism-related factors is dependent on the expression of circadian clock gene *Bmal1* and *Clock*.

**Figure 10:**
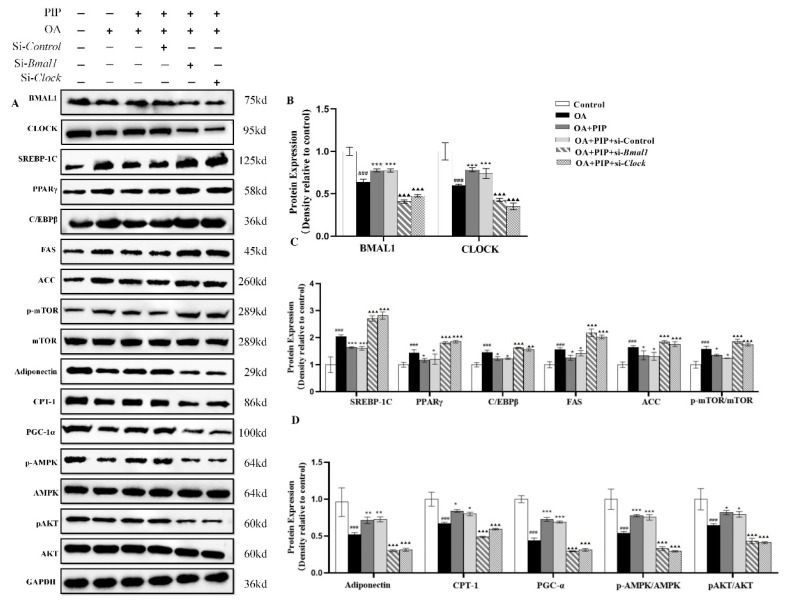
The regulation of circadian clock and lipid metabolism-related factors by PIP in protein levels. HepG2 cells were transfected with the si-*Control* or si-*Bmal1*/si-*Clock* for 24 h; the cultured cells were treated with OA (400 μM) and PIP (50 μM) for 24 h. GAPDH was used as a loading control. (**A**) The expression levels of core circadian clock genes and lipid-related factors in HepG2 cells determined by western blot analysis. (**B**) Protein content of BMAL1 and CLOCK. (**C**) Protein content of SREBP-1C, CEBP/β, PPARγ, FAS, ACC, and p-mTOR/mTOR. (**D**) Protein content of Adiponectin, PGC-1α, CPT-1, p-AMPK/AMPK, and p-AKT/AKT. The densitometric analysis of the blots were expressed as the means ± SD, *n* ≥ 3. ^###^ *p* < 0.001 versus control group, * *p* < 0.05, ** *p* < 0.01, *** *p* < 0.001 versus OA group, ^▲▲^ *p* < 0.01, ^▲▲▲^ *p* < 0.001 versus OA + PIP group.

The protein expression levels of circadian clock genes and lipid metabolism-related factors were also evaluated by western blot. As a central controller of cell growth and proliferation, mTOR could serve with AMPK and AKT as signaling pathways for regulating cellular metabolism, energy homeostasis, and cell growth [39]. Ramanathan et al. also reported a key role of AMPK and mTOR in regulating the rhythmic expression of the core circadian clock genes in the SCN [40,41]. As presented in Figure 10A,D, OA decreased the phosphorylated levels of AMPK and AKT, which were elevated by PIP, indicating that the AMPK/AKT signaling pathway is involved in the regulation effect of PIP on lipid homeostasis [42,43]. The increased phosphorylated level of mTOR by OA was also reduced by PIP, suggesting that PIP attenuated the lipid accumulation by inhibiting the activation of the mTOR signaling pathway [39]. In the si-*Bmal1* and si-*Clock* groups, the restoring effect of PIP on p-AMPK, p-AKT, and p-mTOR was weakened. The results suggested that PIP may prevent lipid accumulation by activating AMPK/AKT-mTOR signaling in a *Bmal1*/*Clock*-dependent manner in HepG2 cells. According to Figure 10, the protein expression levels of circadian clock genes and lipid metabolism-related factors were disrupted in the OA-treated group compared with the control group, but recovered after PIP treatment. This result was consistent with the result in mRNA expression levels, demonstrating that *Bmal1* and *Clock* played an important role in the regulating effect of PIP on OA-induced lipid-metabolism disorders in HepG2 cells.

In this study, HepG2 cells were also transfected with nonspecific scrambled siRNA (OA + PIP + si-*Control* group) for 24 h to evaluate whether transfection reagents could interfere with the regulating effect of PIP on the circadian rhythm and lipid metabolism disorder. Compared with the OA + PIP group, the expression of circadian clock genes and lipid metabolism-related genes showed no significant change in the OA + PIP + si-*Control* group, suggesting that the transfection reagents did not affect the circadian rhythms and lipid metabolism process.

## 3. Materials and Methods

### 3.1. Materials

PIP (purity 98%) was purchased from Xi’an Tianfeng Biotechnology Co., Ltd. (Xi’an, China). OA (purity~99%) was purchased from Shanghai Yuanye Biotechnology Co., Ltd. (Shanghai, China). Milli-Q water (18.3 MΩ) was used in all experiments.

### 3.2. Cell Culture and Viability Assay

HepG2 cells were obtained from Sangon Biotech Co., Ltd. (D611027-0001, Shanghai, China) and cultured in Dulbecco’s modified Eagle medium (DMEM) medium (Gibco, Carlsbad, CA, USA) containing 10% fetal bovine serum (Gibco, Carlsbad, CA, USA), 100 KU/L penicillin (Gibco, Carlsbad, CA, USA) and 100 μg/mL streptomycin (Gibco, Carlsbad, CA, USA). Cells were maintained at 37 °C in a humidified atmosphere of 5% CO_2_. 3-(4,5-dimethyl-2-thiazolyl)-2,5-diphenyl-2-H-tetrazolium bromide (MTT) (purity~98%) was purchased from Macklin Biochemical Co., Ltd. (Shanghai, China). Dimethyl sulfoxide (DMSO) (purity~99%) was purchased from Macklin Biochemical Co., Ltd. (Shanghai, China). Hifair^®^ II 1st Strand cDNA Synthesis SuperMix for qPCR (gDNA digester plus) and Hieff^®^ qPCR SYBR Green Master Mix (Low Rox) were purchased from Yeasen Biotechnology Co., Ltd. (Shanghai, China).

HepG2 cells were incubated overnight in 96-well plates at a density of 1 × 10^6^ cells/well. The cells were cultured with OA and PIP at different concentrations for 24 h. Subsequently, the final concentration of 0.5 mg/mL MTT was added and incubated at 37 °C for 4 h. After removing the MTT from each well, the insoluble formazan crystals were dissolved by DMSO, and the absorbance was measured with a microplate reader (PerkinElmer, Waltham, MA, USA) at 490 nm.

### 3.3. Measurement of Lipid Profiles and Enzyme Activity in HepG2 Cells

The levels of glutathione (GSH), total protein (TP), cholesterol (TC), triglyceride (TG), HDL cholesterol (HDL-C), and LDL cholesterol (LDL-C) in HepG2 cells were measured using enzymatic assay kits (Nanjing Jiancheng Bioengineering Institute, Nanjing, Jiangsu, China). Aspartate aminotransferase (AST), Alanine aminotransferase (ALT), and lactate dehydrogenase (LDH) were detected using enzymatic assay kits (Nanjing Jiancheng Bioengineering Institute, Nanjing, Jiangsu, China).

### 3.4. Transfection of HepG2 Cells

During transfection, the si-*Control* (forward, 5′-UUCUCCGAACGUGUCACGUTT-3′, reverse, 5′-ACGUGACACGUUCGGAGAATT-3′), si-*Bmal1* (forward, 5′-GGUUAUCCAUAUUCUGAUATT-3′, reverse, 5′- UAUCAGAAUAUGGAUAACCTT-3′), or si-*Clock* (5′-GCAACUUGCACCUAUAAAUTT-3′, reverse, 5′-AUUUAUAGGUGCAGUUGCTT-3′) plasmids were transfected into cells cultured in 6-well plates using Lipofectamine 2000 (Invitrogen). After transfection with small interfering RNA (siRNA) for 24 h (transfection efficiency was checked by RT-qPCR), the cells were incubated with OA (400 μM) and PIP (50 μM) for 24 h and then collected for further analysis.

### 3.5. JC-1, DCFDA, and Nile Red Staining

The mitochondrial membrane potential (MMP) was evaluated using the JC-1 assay kit (Nanjing Jiancheng Bioengineering Institute, Nanjing, Jiangsu, China). After treatment, cells were stained with 5 g/mL JC-1 for 30 min at 37 °C in the dark and washed twice with PBS. The cell redox state was detected by a fluorescence microscope (Axio observer A1, Carl Zeiss, Jena, Germany) and measured by PerkinElmer Enspire multimode plate reader (PerkinElmer, Waltham, MA, USA). The levels of MMP were determined by the intensity ratio of green/red fluorescence. The reactive oxygen species (ROS) from HepG2 cells was assayed by reactive oxygen species assay kit (Nanjing Jiancheng Bioengineering Institute). 2′,7′-dichlorofluorescein diacetate (DCFH-DA) (Nanjing Jiancheng Bioengineering Institute) could be converted to the 2′,7′-dichlorofluorescein (DCF), its highly fluorescent oxidation product by intracellular ROS. The DCF fluorescence intensity of HepG2 cells was observed by fluorescence at 495 nm excitation wavelength and 529 nm emission wavelengths.

Cells were stained with 10 μg/mL of Nile red (Macklin Biochemical Co., Ltd., Shanghai, China), a selective fluorescent stain for intracellular lipid droplets. The stained cells were observed under a fluorescent microscope at 543 nm excitation and 598 nm emission wavelengths. Fluorescence was detected using a PerkinElmer Enspire multimode plate reader (PerkinElmer, Waltham, MA, USA).

### 3.6. RNA Isolation and Real-Time qPCR

Total RNA was extracted from HepG2 cells with an extraction kit (TaKaRa, Dalian, China), and cDNA was synthesized using the Primescript RT Master Mix reverse transcription kit (TaKaRa, Dalian, China). The relative mRNA expression was quantified by RT-qPCR using an SYBR green PCR kit (TaKaRa, Dalian, China) and the CFX96 real-time system (Bio-Rad, Hercules, CA, USA). The gene-specific primers used in the cell study are summarized in Table 1. The method of 2^−ΔΔCt^ was used to calculate the relative expression level of target genes, which were normalized to glyceraldehyde-3-phosphate dehydrogenase (GAPDH).

### 3.7. Western Blot Analysis

The treated HepG2 cell lysates were prepared by dissolving in an SDS sample buffer. SDS-PAGE analysis and Western blot detection were performed as described in previous research. [13] Antibodies including anti-BMAL1(14268-1-AP), anti-CLOCK (18094-1-AP), anti-SREBP-1C (14088-1-AP), anti-PPARγ (16643-1-AP), anti-C/EBPβ (66649-1-IG), anti-FAS (66369-1-Ig), anti-ACC (67373-1-Ig), anti-P-mTOR (67778-1-Ig), anti-mTOR (10176-2-AP), adiponectin (21613-1-AP), anti-CPT-1 (15184-1-AP), anti-PGC-1α (20658-1-AP), anti-AMPK (66536-1-Ig), anti-P-AKT (28731-1-AP), and anti-AKT (10176-1-AP) were purchased from Proteintech (Chicago, IL, USA). And the anti-GAPDH (ATPA00013Rb) was purchased from Atagenix (Wuhan, Hubei, China). The anti-P-AMPK (#2535) was purchased from Cell Signaling Technology (Beverly, MA, USA). The primary antibodies for western blot in this study are listed in Table 2. Densitometric analysis of Western blots was performed using Quantity One software (Version 4.6.2, Bio-Rad, Hercules, CA, USA).

### 3.8. Statistical Analysis

Data were presented as means ± standard deviation (SD). Qualitative variables between groups were assesed through one-way analysis of variance (ANOVA) using GraphPad Prism (Version 8.3, GraphPad Software, Inc., San Diego, CA, USA). Differences at *p* < 0.05, 0.01, and 0.001 were considered statistically significant.

## 4. Conclusions

In summary, PIP treatment ameliorated lipid metabolic disturbance, redox status imbalance, mitochondria dysfunction, and the circadian misalignment triggered by OA. Meanwhile, silencing of *Bmal1* and *Clock* gene inhibited the recovery effect of PIP on the lipid-metabolism disorder in HepG2 cells. The underlying mechanism might be that *Bmal1* and *Clock* genes were involved in regulating the expression of lipid metabolism-related factors by PIP in both protein and mRNA levels. Besides, PIP alleviated lipid disorders via strengthening the SREBP-1c/PPARγand AMPK/AKT-mTOR signaling pathways in a *Bmal1*/*Clock*-dependent manner. This study could provide new insights into the application of PIP in the prevention and treatment of obesity-related metabolic disorders through the modulation of circadian clock genes.

## Figures and Tables

**Table 1:** Primer Sequences Used for Quantitative Real-Time PCR Analysis.

Gene	Forward Primer	Reverse Primer
*Bmal1*	ATGGGGCTGGATGAAGACAA	CTGTTGCCCTCTGGTCTACA
*Clock*	ACGACGAGAACTTGGCATTG	GGTGTTGAGGAAGGGTCTGA
*Per1*	AAGTCCGTCTTCTGCCGTAT	TATCCGGGGAGCTTCGTAAC
*Per2*	AGCCGGAGTTAGAGATGGTG	TCTGCTCCTCCTTCTGTGTG
*Cry1*	GTCTACATCCTGGACCCCTG	CTGGGAAACACATCTGCTGG
*Cry2*	GGGAGGAGAGACAGAAGCTC	AATAGGGAGAGGGGAGGTGT
*Reverbα*	CTGGGAGGATTTCTCCATGA	TTCACGTTGAACAACGAAGC
*Srebp-1c*	CTGAGGCAAAGCTGAATAAATCTGCTG	GTTCTCCTGCTTGAGTTTCTGGTTG
*Pparγ*	TGCTGTTATGGGTGAAACTCTG	CTGTGTCAACCATGGTAATTTCTT
*Cebpb*	ACGAGCGCGCCATCGACTTC	GAAGCCCGGCTCCGCCTTG
*Adipoq*	CCTGAACCCTACAAGCGATG	GGTTCCACTTCTTTGTCCTCG
*Fas*	TGCCCAAGTGACTGACATCA	CATCCCCATTGACTGTGCAG
*Acc*	CAACTTTGTGCCCACGGTTA	TTTGTCAGGAAGAGGCGGAT
*Akt*	CTGCCCTTCTACAACCAGGA	ATGATCTCCTTGGCGTCCTC
*mTOR*	CCTGCCTTTGTCATGCCTTT	CTGGGTTTGGATCAGGGTCT
*Pgc-1α*	AGCGCCGTGTGATTTATGTC	TGCGTCCACAAAAGTACAGC
*Cpt-1*	CCTCCGTAGCTGACTCGGTA	GGAGTGACCGTGAACTGAAAG
*Ampk*	GACAAGCCCACCTGATTC	TTCCTTCGTACACGCAAA
*GAPDH*	TCAAGAAGGTGGTGAAGCAGG	TCAAAGGTGGAGGAGTGGGT

**Table 2:** Primary Antibodies for Western Blot in This Study.

Primary Antibodies	Catalogue	Company	Dilution Rates
BMAL1	14268-1-AP	Proteintech	1:600
CLOCK	18094-1-AP	Proteintech	1:600
SREBP-1C	14088-1-AP	Proteintech	1:1000
PPARγ	16643-1-AP	Proteintech	1:1000
C/EBPβ	66649-1-IG	Proteintech	1:2000
FAS	66369-1-Ig	Proteintech	1:5000
ACC	67373-1-Ig	Proteintech	1:5000
p-mTOR	67778-1-Ig	Proteintech	1:1000
mTOR	10176-2-AP	Proteintech	1:1000
Adiponectin	21613-1-AP	Proteintech	1:1000
CPT-1	15184-1-AP	Proteintech	1:5000
PGC-1α	20658-1-AP	Proteintech	1:1000
p-AMPK	#2535	CST	1:1000
AMPK	66536-1-Ig	Proteintech	1:1000
p-AKT	28731-1-AP	Proteintech	1:1000
AKT	10176-1-AP	Proteintech	1:1000
GAPDH	ATPA00013Rb	Atagenix	1:5000

## Data Availability

The HepG2 cell line was obtained from Sangon Biotech Co., Ltd. (D611027-0001, Shanghai, China).

## Abbreviations

ACC	acetyl coenzyme A carboxylase
ALT	alanine aminotransferase
AMPK	AMP-activated protein kinase
AST	aspartate aminotransferase
BMAL1	aryl hydrocarbon receptor nuclear translocatorlike 1 proteins
*Bmal1*	aryl hydrocarbon receptor nuclear translocatorlike 1
C/EBPβ	adipogenesis-associated transcription factor CCAAT enhancer binding proteins β
*Ccgs*	other clock-controlled genes
CLOCK	circadian locomotor output cycles kaput proteins
*Clock*	circadian locomotor output cycles kaput
CPT-1	carnitine palmitoyl transferase
CRYs	cryptochrome proteins
*Crys*	cryptochrome genes 1/2
DCF	dichlorofluorescin
DCFH-DA	2,7-dichlorofluorescin diacetate
FAS	fatty acid synthase
HDL-C	HDL cholesterol
LDH	lactate dehydrogenase
LDL-C	LDL cholesterol
MMP	mitochondrial membrane potential
mTOR	mammalian/mechanistic target of rapamycin
NAD^+^	nicotinamide adenine dinucleotide
Nampt	nicotinamide phosphoribosyltransferase gene
OA	oleic acid
PERs	period proteins
Pers	period genes 1/2/3
PGC-1α	peroxlsome proliferator-activated receptor-γ coactlvator-1α
PPARs	peroxisome proliferator-activated receptors
PPARγ	peroxisome proliferator activated receptor gamma
Reverbs	reverse erythroblastosis virus α/β
RORE	ROR response element
RORs	retinoic acid-related orphan receptors
ROS	reactive oxygen species
SCN	suprachiasmatic nucleus
SIRT1	silent information regulator 1
SREBP-1c	sterol-regulatory-element-binding protein 1c
TC	total cholesterol
TG	total triglyceride
TP	total protein
TTFL	transcriptional/translational feedback loop
